# Effect of dietary management on the gastric endocrine cells in patients with irritable bowel syndrome

**DOI:** 10.1038/ejcn.2014.151

**Published:** 2014-08-06

**Authors:** T Mazzawi, T Hausken, D Gundersen, M El-Salhy

**Affiliations:** 1 Section for Gastroenterology, Department of Medicine, Stord Hospital, Stord, Norway; 2 Section for Gastroenterology, Institute of Medicine, Bergen University, Bergen, Norway; 3 Department of Research, Helse-Fonna, Haugesund, Norway

## Abstract

**Background/objectives:**

The gastric endocrine cells in patients with irritable bowel syndrome (IBS) tend to normalize following dietary guidance. The aim of the present study was to identify the gastric endocrine cell types that are changed following such dietary guidance.

**Subjects/methods:**

Fourteen IBS patients and 14 healthy subjects were included in the study. Patients received three sessions of individual dietary management guidance. Gastroscopy was performed on both the controls and the patients at baseline and then again for the patients at 3–9 months after dietary guidance. Biopsy samples from the corpus and antrum were immunostained for all gastric endocrine cell types. Endocrine cells were quantified by computerized image analysis.

**Results:**

The densities of the ghrelin cells for the controls and IBS patients before and after dietary guidance were 149.6±36.2 (mean±s.e.m.; 95% confidence interval (CI) 71.3–227.8), 114.5±32.7 and 161.8±37.8 cells/mm^2^, respectively. The densities of the gastrin cells in these groups were 155.8±21.0 (95% CI 110.3–201.2), 159.4±24.3 and 211.6±28.0 cells/mm^2^, respectively; the corresponding densities of serotonin cells in the corpus were 18.2±3.9 (95% CI 9.8–26.6), 10.6±3.4 and 14±2.0 cells/mm^2^ and in the antrum were 44.6±12.2 (95% CI 18.1–71.1), 1.7±0.5 and 14.7±6.3 cells/mm^2^. The densities of the somatostatin cells in the corpus were 40.0±7.7 (95% CI 23.5–56.5), 23.0±3.0 and 37.3±4.2 cells/mm^2^, respectively, and in the antrum were 138.9±22.0 (95% CI 91.4–186.3), 95.6±15.9 and 86.0±16.9 cells/mm^2^, respectively.

**Conclusions:**

The densities of all of the gastric endocrine cell types changed towards the healthy control values in the IBS patients following a change in food intake.

## Introduction

The gastrointestinal endocrine cells regulate several functions of the gut such as sensation, motility, secretion, absorption, local immune defence and food intake via controlling the appetite.^1,2^ These cells have specialized microvilli that project into the gut lumen and act as sensors for the luminal contents, mainly nutrients, and respond to luminal stimuli by releasing hormones that generally target other parts of the digestive system.^1,2^

Abnormalities in the gastrointestinal cells have been reported in patients with irritable bowel syndrome (IBS), and it has been suggested that this abnormality has a major role in the pathophysiology of IBS.^1,2^ It has also been demonstrated that the gastric endocrine cells are abnormal in IBS patients, and the conclusion has been drawn that the pathophysiology of IBS is not restricted to the large intestine.^1, 2, 3, 4^

A previous study performed by our group found that a change in food intake in IBS patients established by dietary guidance reduced the symptoms and improved the quality of life in these patients.^5^ Investigation of the total gastric endocrine cell population, as revealed by chromogranin A staining, revealed that in the same patient cohort the density of gastric endocrine cells tended to normalize following dietary guidance.^6^ The aim of the present study was to identify which gastric endocrine cell types were affected by a change in food intake in that same cohort of IBS patients.^5,6^

## Materials and methods

### Patients and controls

The inclusion criteria for this study were that patients fulfilled Rome-III criteria for IBS diagnosis and were aged 18–70 years. The following exclusion criteria were applied: women who were pregnant or lactating, or who had a Caesarean section or hysterectomy, and patients with organic gastrointestinal or other systemic diseases, drug abuse, serious psychiatric disturbances or previous abdominal surgery (with the exception of appendectomy). Eight patients did not consume any kind of medications. Six patients used one or a combination of the following: proton pump inhibitors (*n*=4), anti-hypertensive Losartan (*n*=1), anti-allergic medications (*n*=3), contraceptive pills (*n*=2), thyroxin substitution tablet (*n*=2), asthma inhalator medications (*n*=1), and anti-depressants/anxiolytics (*n*=2). These patients were told not to take any kind proton pump inhibitors during the study.

Patients who underwent gastroscopy to investigate gastrointestinal bleeding, where the source of bleeding was identified as haemorrhoids (*n*=3) or angiodysplasia (*n*=1) or because of health worries in healthy subjects due to family member(s) having been diagnosed with gastrointestinal cancer (*n*=10) were used as controls. The control group comprised nine females and five males with a mean age of 54 years (range 26–70 years).

The study was performed in accordance with the Declaration of Helsinki and approved by the local Committee for Medical Research Ethics, West, Bergen, Norway. All patients submitted both oral and written consent to participate.

### Study design

A total of 46 patients were recruited to the study. They were 35 females and 11 males with a mean age of 35 years (range 18–69 years). Patients were examined physically, and blood tests were taken to exclude inflammation, infection or other organic disease. These patients also underwent colonoscopy with segmental biopsy sampling to exclude microscopic colitis. Each patient was scheduled for three sessions of individual dietary management guidance of about 45 min, each with a nurse with education and long experience in dietary guidance in IBS. These sessions were timed at intervals of at least 2 weeks. The patients underwent gastroscopy, before the first session and 3–9 months (median 4 months) following the third dietary management guidance session.

### Individual dietary management guidance

The information was delivered orally with the help of charts, as well as written illustrated information. The first session included general information about IBS and the importance of regular and healthy eating habits, the diets that worsen IBS symptoms, such as insoluble dietary fibre and FODMAPs (the poorly absorbed highly fermentable oligosaccharides, disaccharides, monosaccharides and polyols). The patients were encouraged to consume dairy products daily and were informed that milk and dairy products do not provoke IBS symptoms. The patients were required to keep a diary for 2 weeks, in which they registered their daily food and drink intake as well as the frequency and degree of abdominal pain, abdominal distension and stool frequency and consistency. In addition, the patients were asked to test a protein-, fat- or carbohydrate-rich/poor diet. The patients were also instructed to avoid the intake of food items supplemented with probiotics, antibiotics and other medications (if not otherwise advised) during the course of the study. In the second session, the information given at the first session was briefly repeated. The nurse and the patient determined the symptom-triggering items based on the information noted in the patient's diary. The patients were then advised to alter the proportions of protein, fat and carbohydrate, avoid items rich in FODMAPs and insoluble fibre, as well as consume vegetables and fruits containing less FODMAPs and insoluble fibre. During the final session, patients described their experience of dietary management to the nurse. The nurse and patient then together designed a suitable diet for the patient, who followed it strictly until the end point of the study.

### Dietary assessment

Dietary intake was assessed using a semi-quantitative, self-administered food frequency questionnaire (MoBa food frequency questionnaire). The questionnaire reported the frequency of consumption and portion size of a series of foods and beverages over a defined period of time. The data were then analysed by software for nutrient calculations. The MoBa food frequency questionnaire used in the current study was developed and validated by the Norwegian Institute of Public Health in Oslo, Norway.^7,8^ It enquires about the intake of 225 food items and captures the dietary habits of the participant, including the intake of any oral supplements, according to typical Norwegian meal patterns. The participants received two sets of MoBa food frequency questionnaire, which were filled out and delivered before and at least 3 months after dietary management guidance.^9^

### Gastroscopy, tissue sampling, histopathology and immunohistochemistry

After an overnight fast, patients and control subjects underwent standard gastroscopy. Four biopsy samples were taken from the corpus (major curvature) and another four biopsy samples from the antrum of the stomach. A further four biopsy samples were taken from the duodenum to exclude coeliac disease.

The biopsy samples were fixed in 4% buffered paraformaldehyde overnight, embedded in paraffin wax and then cut into 5-μm-thick sections. Biopsy samples from the stomach and duodenum were examined histopathologically. Biopsy samples from the corpus and antrum were stained with haematoxylin–eosin and immunostained using the avidin–biotin complex method using the Vectastain ABC kit (Vector Laboratories, Burlingame, CA, USA) and the 3,3′-diaminobenzidine peroxidase substrate kit (Vector Laboratories) as described previously.^10^ Briefly, the sections were incubated for 2 h at room temperature with a primary antibody/antiserum, after which they were washed in phosphate-buffered saline (pH 7.4) and incubated for 30 min at room temperature with biotinylated swine antimouse immunoglobulin G diluted to 1:200. The slides were washed with phosphate-buffered saline and then incubated for 30 min with avidin–biotin–peroxidase complex diluted to 1:100. The slides were again washed with phosphate-buffered saline, submerged in 3,3′-diaminobenzidine peroxidase substrate and then counterstained with haematoxylin–eosin. The primary antibodies/antisera used were monoclonal mouse anti-N-terminal of human ghrelin (code 2016003, Millipore, Temecula, CA, USA), monoclonal mouse antihuman serotonin (clone 5HT-H209, code M0758, Dako, Glostrup, Denmark), polyclonal rabbit antisynthetic, cyclic somatostatin (code A0566, Dako), monoclonal mouse antihistamine (serotonin)-hexamethylene, diisocyanate-bovine serum albumin (code 2273835, Millipore, Darmstadt, Germany) and polyclonal rabbit antihuman gastrin (code A0568, Dako).

### Computerized image analysis

The densities of the various types of endocrine cells in the corpus and antrum in patients with IBS and controls were evaluated using the software Cell ⁁D (Olympus, Tokyo, Japan). The number of endocrine cells and the area of epithelial cells were measured in 10 randomly chosen fields, at a magnification of × 40, for which each field represented a tissue area of 0.14 mm^2^. The endocrine cell density is expressed as the number of cells per square millimetre of epithelium. Quantification was conducted by the same person (TM), who was blinded to the identity of the sections.

### Statistical analysis

The paired *t*-test was used to compare patients before and after dietary guidance and healthy controls. The data are presented as mean±s.e.m. values. The cutoff for statistical significance was set at *P*<0.05.

## Results

### Patients and controls

Twenty-five patients withdrew their consent, 2 before and 23 after receiving dietary guidance and with consequent improvement in their symptoms. Two patients were excluded because of non-compliance, two because they were diagnosed with coeliac disease and one who had been diagnosed with lupus. In addition, two patients were excluded because of pregnancy during the study and moving abroad. Thus, 14 of the initial 46 patients completed the entire study. These patients comprised 9 females and 5 males with a mean age of 34 years (range 20–45 years). Biopsy samples were not obtained from the corpus of one of the female patients.

### Dietary assessment

The daily total consumption (mean±s.e.m.) of FODMAP-rich fruits and vegetables decreased significantly after dietary guidance compared with before receiving dietary guidance (9.2±3.2, 16.2±5.3 g, respectively, *P*=0.016).^9^ The daily consumption of fibre (mean±s.e.m.) before and after dietary guidance were 27.4±2.5 and 23.1±2.2 g, respectively, (*P*=0.093).^9^

### Gastroscopy, histopathology and immunohistochemistry

Endoscopy of the upper oesophagus, stomach and duodenum revealed a normal appearance, and histopathological examinations of the stomach and duodenum revealed a normal structure. Serotonin- and somatostatin-immunoreactive cells were found in the mucosa of both the corpus and antrum in the patients and controls. Whereas ghrelin-positive cells were found exclusively in the oxyntic mucosa of the corpus, gastrin-positive cells were found only in the antrum. The number of histamine-immunoreactive cells in the biopsy samples used in the study was low and did not allow reliable quantification.

### Computerized image analyses

#### Ghrelin

**Figure 1:**
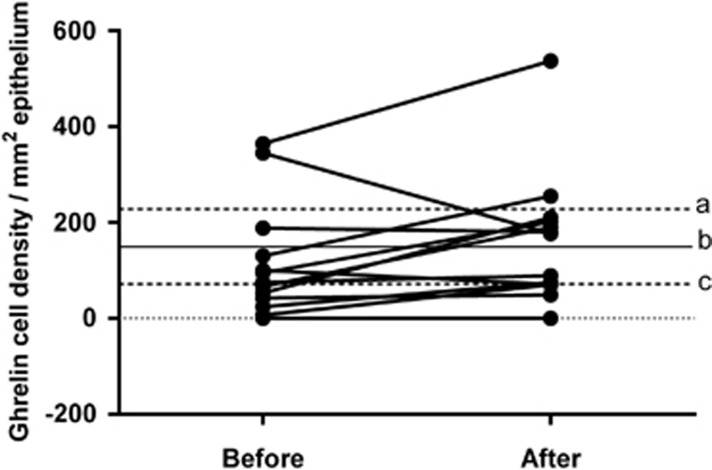
Ghrelin cell density in the oxyntic mucosa in controls and in IBS patients before and after receiving dietary guidance. The dashed lines labelled ‘a' and ‘c' show the upper and lower limits of the 95% CI in healthy controls, respectively, while line ‘b' shows the mean ghrelin cell density.

**Figure 2:**
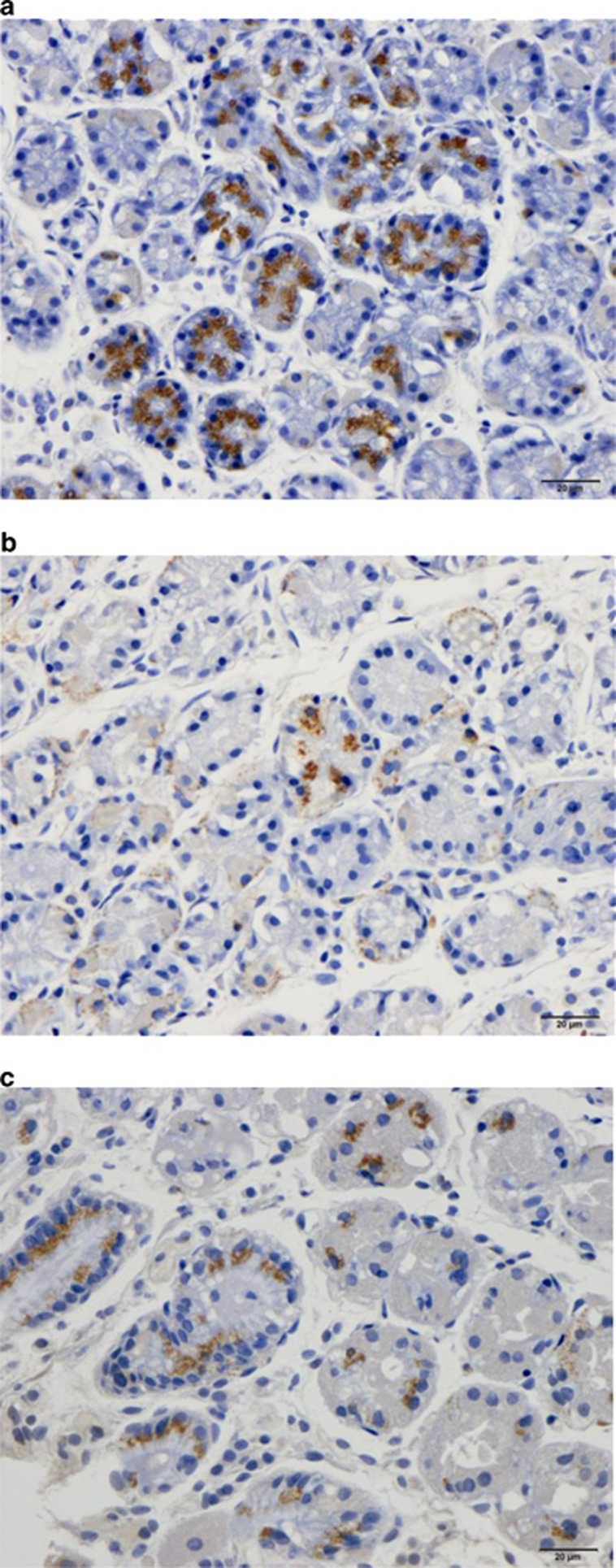
Ghrelin-immunoreactive cells in the oxyntic mucosa in a control (**a**) and in an IBS patient before (**b**) and after (**c**) receiving dietary guidance.

In the control group, the density of ghrelin cells in the gastric corpus was 149.6±36.2 cells/mm^2^ (95% confidence interval (CI) 71.3–227.8). The density of ghrelin cells in the IBS patients did not differ significantly between before (114.5±32.7 cells/mm^2^) and after (161.8±37.8 cells/mm^2^) receiving dietary guidance (*P=*0.09, paired *t*-test; Figures 1 and 2).

#### Gastrin

**Figure 3:**
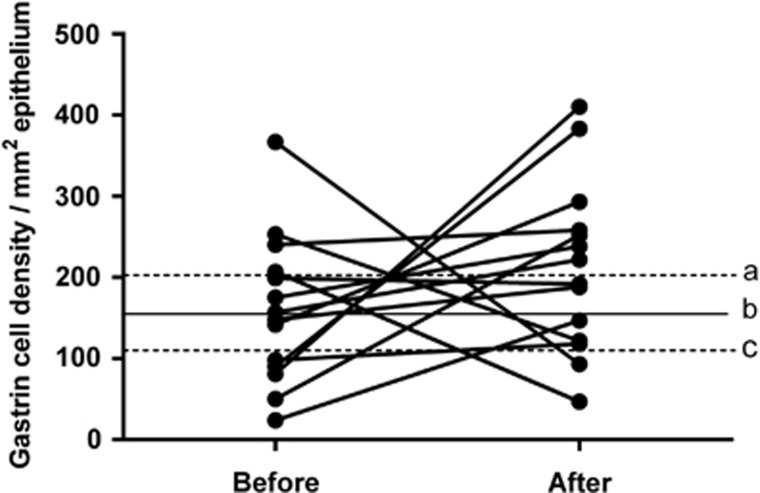
Gastrin cell density in the antrum in controls and in IBS patients before and after receiving dietary guidance. The symbols are the same as in Figure 1.

The densities of the gastrin cells in the antrum were 155.8±21.0 (95% CI 110.3–201.2), 159.4±24.3 and 211.6±28.0 cells/mm^2^ in the controls and in the IBS patients before and after dietary guidance, respectively (Figure 3). The cell density did not differ significantly between before and after dietary guidance in the IBS patients (*P=*0.26).

#### Serotonin

**Figure 4:**
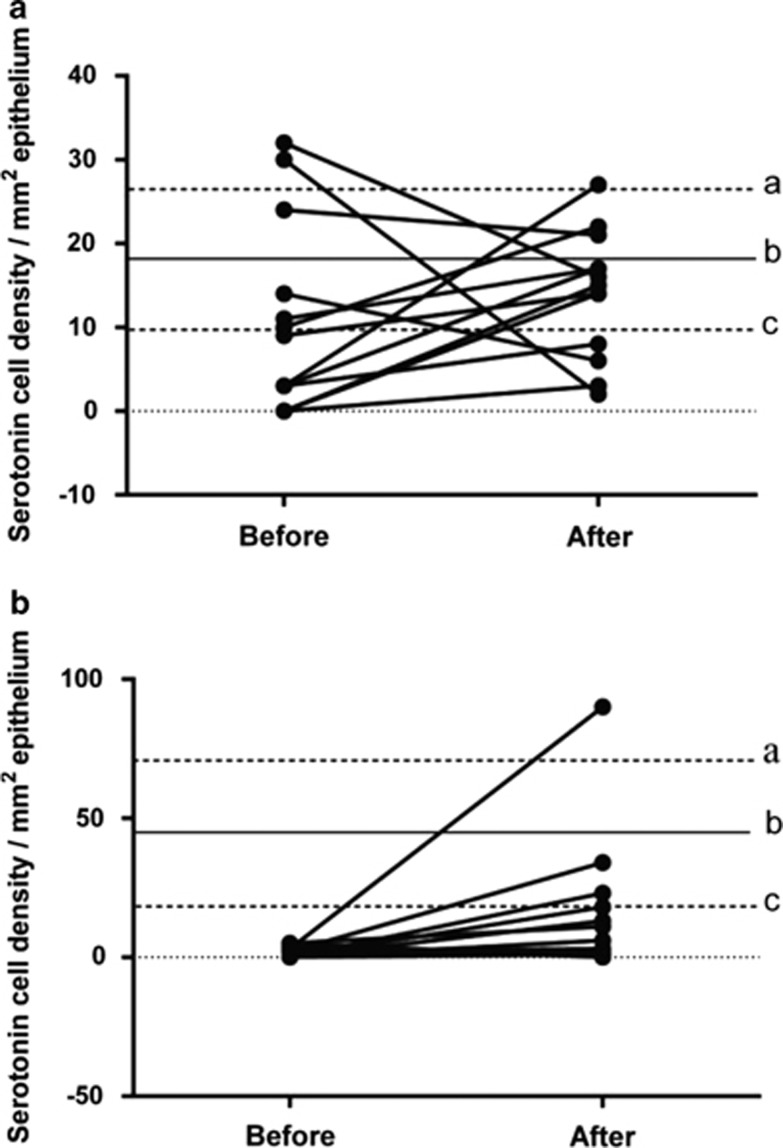
Serotonin cell density in the oxyntic mucosa (**a**) and the antrum (**b**) in controls and in IBS patients before and after receiving dietary guidance. The symbols are the same as in Figure 1.

In the corpus, the density of serotonin cells in the control group was 18.2±3.9 cells/mm^2^ (95% CI 9.8, 26.6), while those in IBS patients before and after receiving dietary guidance were 10.6±3.4 and 14.0±2.0 cells/mm^2^, respectively (Figure 4). The cell density did not differ significantly between before and after dietary guidance (*P*=0.4). In the antrum, the corresponding values were 44.6±12.2 cells/mm^2^ (95% CI 18.1–71.1), 1.7±0.5 and 14.7±6.3 cells/mm^2^, respectively. There was no significant difference between the findings before and after dietary guidance in the IBS patients (*P*=0.06).

#### Somatostatin

**Figure 5:**
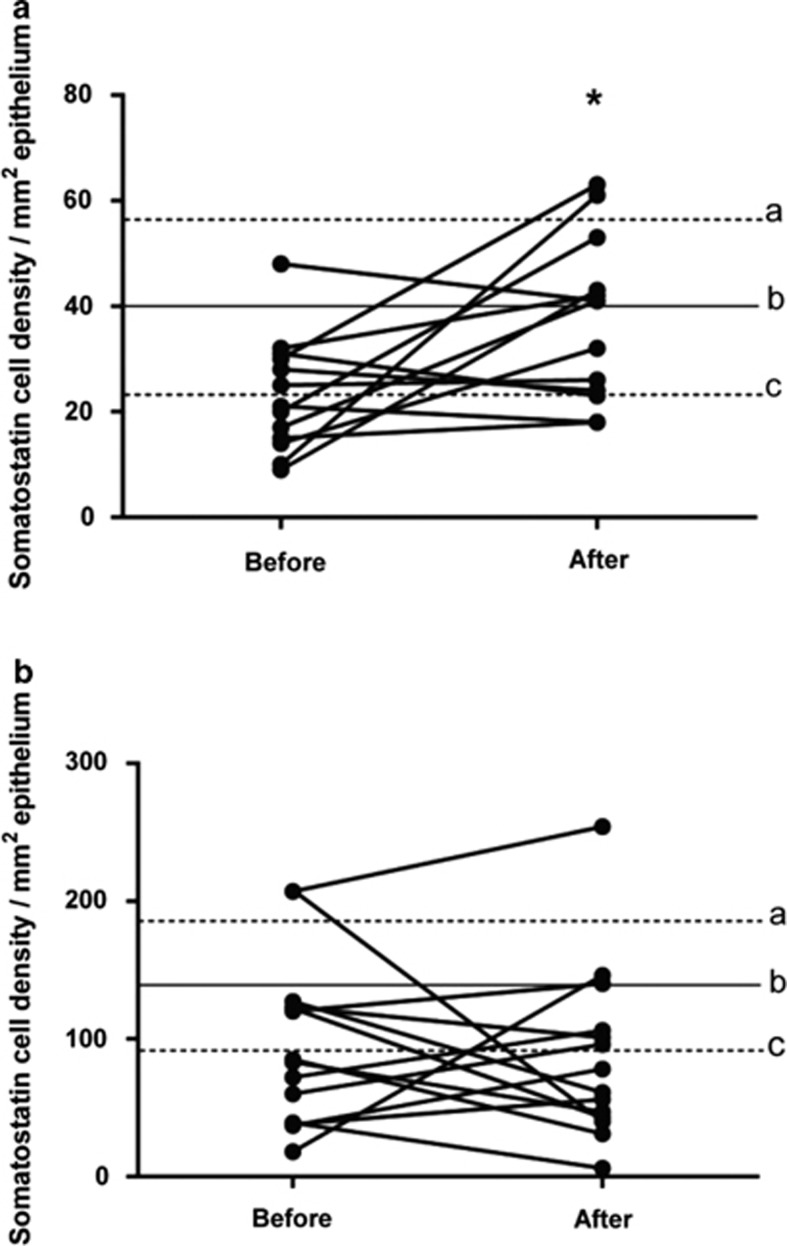
Somatostatin cell density in the oxyntic mucosa (**a**) and the antrum (**b**) in controls and in IBS patients before and after receiving dietary guidance. The symbols are the same as in Figure 1. *P<0.05.

**Figure 6:**
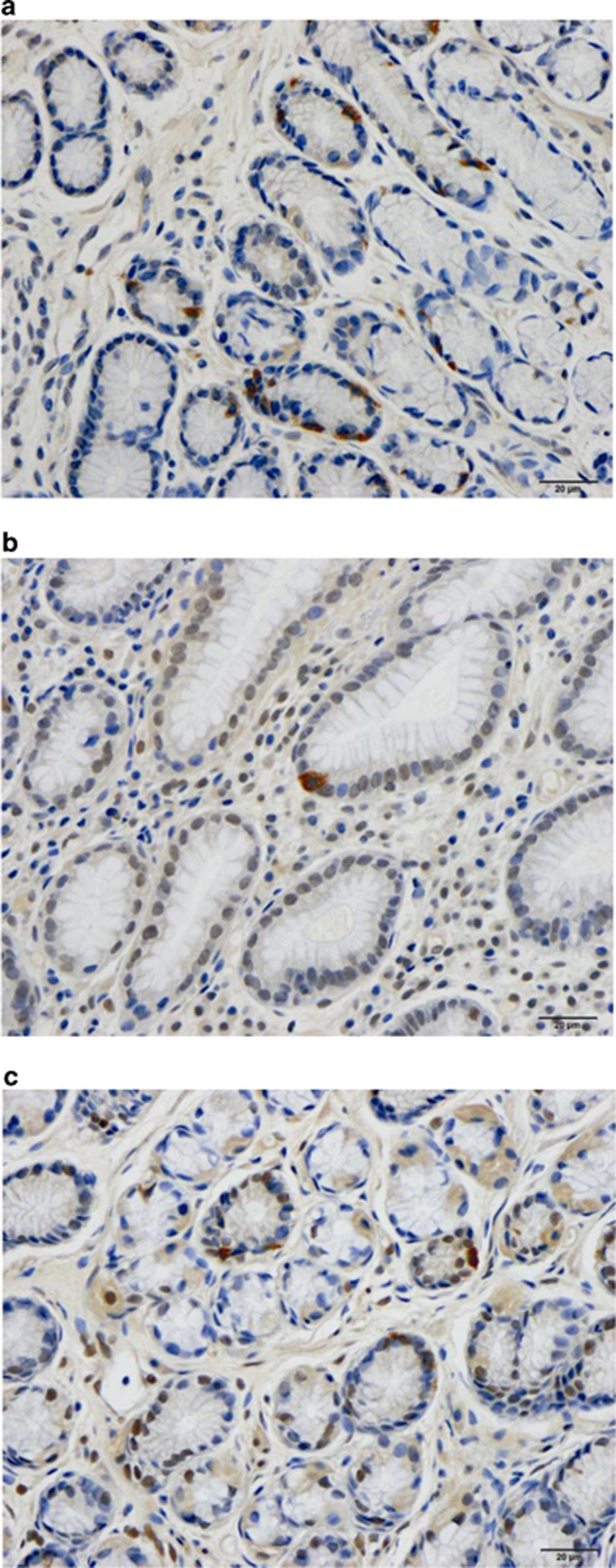
Somatostatin-immunoreactive cells in the gastric antrum in a control (**a**) and in an IBS patient before (**b**) and after (**c**) receiving dietary guidance.

In the control group, the densities of somatostatin cells in the corpus and antrum were 40.0±7.7 cells/mm^2^ (95% CI 23.5–56.5) and 138.9±22.0 cells/mm^2^ (95% CI 91.4–186.3), respectively. The somatostatin cell density in the antrum in the IBS patients did not differ significantly between before and after receiving dietary guidance (95.6±15.9 and 86±16.9 cells/mm^2^, respectively; *P*=0.6). However, the cell density was significantly higher in the corpus compared with before the intervention (23.0±3.0 and 37.3±4.2 cells/mm^2^, respectively; *P*=0.02; Figures 5 and 6).

## Discussion

Studies of IBS patients have experienced high dropout rates, ranging from 44% to 48%.^11,12^ The dropout rate in the present study was also high, at 54%, which may be explained by the design of the study, which was demanding, involving two invasive examinations (gastroscopy) and the expectation that the patient follow the recommended diet strictly for at least 3 months. It is noteworthy that the dropouts occurred mostly after receiving dietary guidance and the associated improvements in the patients' symptoms. There were also other factors that decreased the number of IBS patients who completed this study. Despite the small number of patients in the studied sample, a clear change in the densities of all the gastric endocrine cells occurred following changes in the food intake in the IBS patients towards the values measured for the healthy control subjects.

The endocrine cells in the oxyntic mucosa of the stomach are the main source of ghrelin in the body. Ghrelin has several functions, including the release of growth hormone and regulating appetite and energy metabolism.^13, 14, 15, 16^ Furthermore, ghrelin accelerates gastric and small- and large-intestinal motility and stimulates the secretion of gastric acid.^17, 18, 19, 20, 21, 22, 23, 24, 25, 26, 27, 28^ Serotonin stimulates large-intestinal motility and accelerates transit through the small and large intestines.^29, 30, 31, 32, 33, 34, 35, 36, 37^ It also activates the submucosal sensory branch of the enteric nerve, which conveys sensation from the gut to the central nervous system.^29,33, 34, 35^ Somatostatin inhibits intestinal contraction and gastrointestinal exocrine and endocrine secretion.^38,39^ Finally, gastrin is the main hormonal stimulant of acid secretion in the stomach.^40, 41, 42, 43, 44^ Given the collective functions of these four types of gastric endocrine cells, it is possible to speculate that the changes in the densities of these cells towards normal levels following a change in food intake are responsible for the simultaneous improvements in IBS symptoms associated with disturbed gut motility, visceral hypersensitivity and abnormal gastrointestinal secretion.

The gut luminal contents, and especially the nutrients, are the main stimuli that trigger gut endocrine cells' activity.^1,2^ Stem cells rapidly differentiate into endocrine cells in the gut, over a period of just a few days.^42,43^ It is tempting to speculate that the change in diet and the consequent change in the contents of the gut lumen are responsible for the changes in the gastric endocrine cells observed here. If this assumption is proven to be true, this would open new frontiers with important clinical implications for IBS patients.
